# Determinants of the Hesitancy toward COVID-19 Vaccination in Eastern European Countries and the Relationship with Health and Vaccine Literacy: A Literature Review

**DOI:** 10.3390/vaccines10050672

**Published:** 2022-04-23

**Authors:** Alina Delia Popa, Armand Iustinian Enache, Iolanda Valentina Popa, Sabina Antonela Antoniu, Raluca Alina Dragomir, Alexandru Burlacu

**Affiliations:** 1 Faculty of Medicine, University of Medicine and Pharmacy “Grigore T Popa”, 700115 Iasi, Romania; alina.popa@umfiasi.ro (A.D.P.); iustinian-armand.enache@umfiasi.ro (A.I.E.); sabina.antoniu@umfiasi.ro (S.A.A.); raluca.dragomir@umfiasi.ro (R.A.D.); alexandru.burlacu@umfiasi.ro (A.B.); 2 Clinic of Pulmonary Diseases, 700115 Iasi, Romania; 3 Institute of Cardiovascular Diseases “Prof. Dr. George I.M. Georgescu”, 700503 Iasi, Romania

**Keywords:** COVID-19, SARS-CoV-2, vaccine hesitancy, vaccination rate, Eastern Europe

## Abstract

Herd immunity is necessary to control the coronavirus disease 2019 (COVID-19) pandemic. However, a low proportion of vaccinated people and low levels of vaccine acceptance have been noted in Eastern Europe. Our paper aimed to review the central attitudes associated with the hesitancy toward COVID-19 vaccination specific to Eastern European countries. The main Eastern European determinants of COVID-19 vaccine acceptance identified from the included studies are: public confidence in the vaccines’ safety and efficacy, vaccine literacy, and public trust in the government and the medical system. Each of these determinants is discussed along with possible improvement measures. Variables specific to Eastern Europe that predict the willingness to vaccinate have also been highlighted. The specific attitudes and their context as identified by our review should be incorporated into local public health programs, with the ultimate goal of reducing viral spreading, mutation emergence, and COVID-19 morbidity and mortality both within the borders of Eastern Europe and beyond.

## 1. Introduction

Even though the World Health Organization (WHO) announced the pandemic more than two years ago, there is no widely available therapy for coronavirus disease 2019 (COVID-19). Paxlovid (nirmatrelvir and ritonavir) tablets, co-packaged for oral use, is approved for the treatment of mild-to-moderate coronavirus disease (COVID-19) in adults and pediatric patients, with positive outcomes especially in those at high risk for progression to severe COVID-19 [1]. Paxlovid significantly reduced the proportion of people with COVID-19–related hospitalization and those who died from any cause by 88% compared to placebo among patients treated within five days of symptom onset [1]. However, this drug is not widely available. According to current knowledge, vaccination of the general population is the only way to avert severe forms of disease and death on a global scale.

Vaccines currently available on the market proved to be effective against hospitalization and death for all SARS-CoV-2 variants up to the Delta [2,3]. Both Moderna (mRNA-1273; Cambridge, MA, USA) and Pfizer BioNTech (BNT162b2; Mainz, Germany) COVID-19 vaccines offer protection greater than 90% [4,5], while Johnson & Johnson (Ad26.CoV2-S; Leiden, The Netherlands) and AstraZeneca-Oxford (AZD1222; Oxford, UK) COVID-19 vaccines offered moderate protection (66% and, respectively, 55–81%) [6,7]. These findings are strengthened by evidence showing a sharp decrease in cases and hospitalizations by 77% and 68%, respectively, within the same timeline in England and Israel [8]. Moreover, an increased proportion of vaccinated people was accompanied by the flattening of the epidemic curve [9,10].

**Figure 1:**
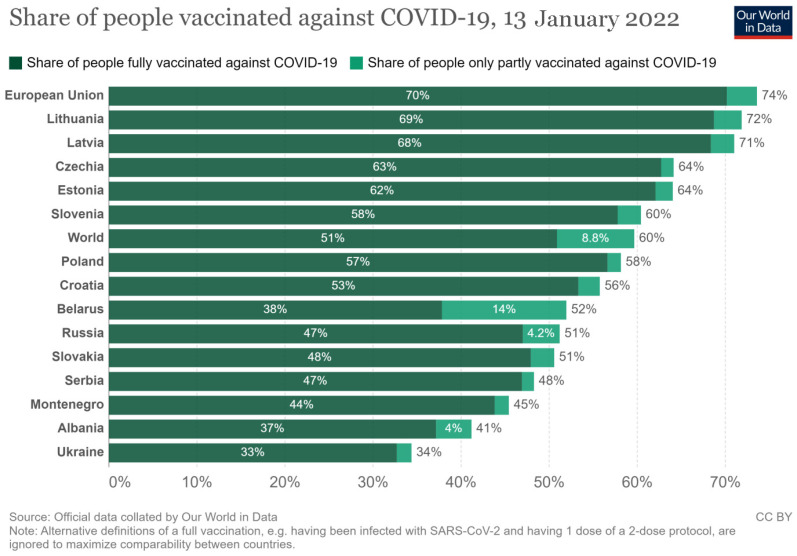
Share of people vaccinated against COVID-19 in Eastern European countries as of 13 January 2022 (after “Our World in Data,” reproduced with permission) [13].

Mathematical models show that if the COVID-19 vaccine is 80% effective, the coverage must be at least 75% in the general population to achieve herd immunity and control the pandemic [11]. Unfortunately, attaining herd immunity is a significant challenge for current healthcare systems worldwide, and the prevalence of hesitancy toward COVID-19 remains high, especially in Eastern Europe [12]. Worldwide, 59.6% of the population was reported to be vaccinated with at least one dose of COVID-19 vaccine as of January 2022 [13]. In the European Union, fully vaccinated people are 70%, but a much lower proportion was noted in Eastern European countries, Figure 1 [13]. 

Eastern Europe ranks first in the continent among subregions ranked by population, having currently 292,309,880 inhabitants, based on the latest United Nations estimates [14]. At the same time as Eastern European countries battle vaccine hesitancy, the region was proved to have the highest adjusted mortality trend ratios [15]. Moreover, the negative implications cross the Eastern Europe borders. In the absence of mass vaccination, besides a high spreading rate among unvaccinated, there is an increased risk of new COVID-19 mutations emergence [16], with new variants emerging out of the east and menacing to discharge themselves even on vaccinated people in the west.

Several reviews dealing with worldwide attitudes and hesitancy toward COVID-19 vaccination have been published [12,17]. However, none addressed primarily and exclusively the determinants of vaccine reticence in Eastern Europe, where a lower overall proportion of vaccine acceptance has been reported. Increasing the vaccination rate is crucial in combating the COVID-19 pandemic, but it requires prior identification of the underlying causes and specific determinants of hesitancy toward vaccines specific to this region.

Our paper aims to review the central attitudes and factors associated with hesitancy toward COVID-19 vaccination specific to Eastern European countries. These specific attitudes must be recognized and incorporated into local public health programs, with the ultimate goal of reducing viral spreading, mutation emergence, and COVID-19 morbidity and mortality both within the borders of Eastern Europe and beyond.

## 2. Materials and Methods

This review was conducted according to the Extension of the Preferred Reporting Items for Systematic Reviews and Meta-Analyses (PRISMA) checklist [18].

### 2.1. Eligibility Criteria

Original research studies published in English addressing attitudes toward COVID-19 vaccination in Eastern European (Belarus, Bulgaria, Czechia, Hungary, Poland, Republic of Moldova, Romania, Russian Federation, Slovakia, and Ukraine) and Southern European ex-communist countries (Albania, Bosnia and Herzegovina, Croatia, Serbia, North Macedonia, and Slovenia) during 2020–2021 were considered eligible for inclusion.

### 2.2. Information Sources and Search

We searched PubMed/Medline and Google Scholar. The following search string was used: (“COVID-19” OR “SARS-CoV-2” OR “nCOV”) AND (“vaccine” OR “vaccination” OR “Pfizer” OR “Moderna” OR “AstraZeneca” OR “BnT162b2” OR “mRNA1273” OR “mRNA” OR “Sputnik” OR “AZD1222” OR “Ad26.CoV2-S”) AND (“attitude” OR “hesitancy” OR “knowledge” OR “willingness” OR “reluctance” OR “reticence” OR “acceptance” OR “opposition” OR “refusal”) AND (“Eastern Europe” OR “Bulgaria” OR “Romania” OR “Ukraine” OR “Serbia” OR “Czech Republic” OR “Poland” OR “Bosnia and Herzegovina” OR “Russia” OR “Slovenia” OR “Slovakia” OR “Croatia” OR “Hungary” OR “Republic of Moldova” OR “Albania” OR “North Macedonia”).

### 2.3. Data Charting Process

Two researchers realized the search, inclusion, and coding of the studies independently. Disagreements were solved by a third senior researcher. Finally, the remaining disagreements were solved by consensus.

### 2.4. Selection of Sources of Evidence

We initially reviewed 2652 studies (1297 on PubMed/Medline and 1355 on Google Scholar) conducted between December 2020 and January 2022. After excluding duplicate titles, 2021 studies conducted worldwide remained. Of these, 517 were conducted in Europe, and among these, 223 were conducted in Eastern European countries. After excluding reviews, reports, current opinions, and studies irrelevant to our objectives, we selected 44 cross-sectional studies that address the motivation for hesitance toward COVID-19 vaccination.

**Figure 2:**
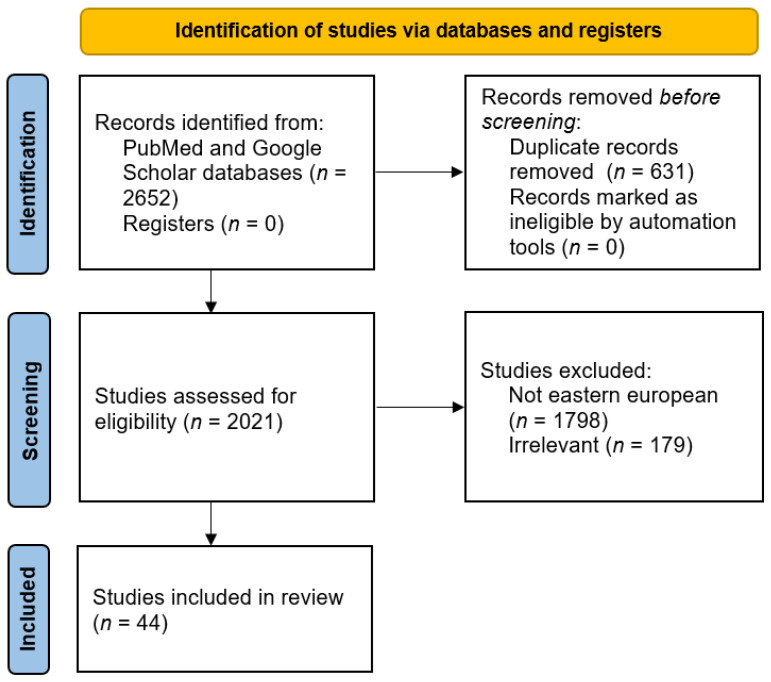
Study selection process and number of papers included.

The study selection process and the number of papers identified in each phase are illustrated in the flowchart (Figure 2). All included studies are coded in Table S1.

Among the included studies, 20 were addressed to the general population, 5 to patients with a chronic illness (4 in cancer patients, one in patients with epilepsy), 9 in healthcare workers, and 5 in medical and nursing students. Among the studies in the general population, eight were conducted in 2020 to investigate the proportion of people willing to get a vaccine and ten during the first four months from the initiation of vaccination programs. Raciborski et al. [19] analyzed the evolution of vaccine acceptance during the fourth wave of the pandemic.

The design of these studies was mainly cross-sectional; only two studies were longitudinal surveys. The preferred method of investigation was the distribution of online standardized questionnaires by email, WhatsApp, or Facebook. Two studies were qualitative using categorical thematic analysis.

**Figure 3:**
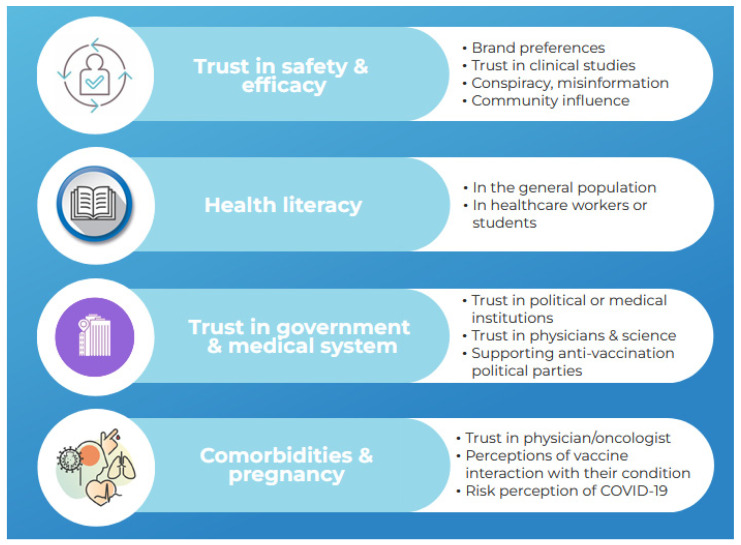
The major Eastern European factors influencing COVID-19 vaccine acceptance.

The main Eastern European determinants of COVID-19 vaccine acceptance identified from the retained studies are public confidence in the vaccines’ safety and efficacy, health and vaccine literacy, and public trust in the government and medical system (Figure 3).

Each of these determinants is discussed below, along with possible improvement measures. Variables specific to Eastern Europe that predict the willingness to vaccinate have also been highlighted. Several studies explored the hesitancy toward COVID-19 vaccination in Eastern European populations with comorbidities or pregnancy [20,21,22,23,24,25,26].

### 2.5. Quality Appraisal

We did not assess the quality or risk of bias of the included studies, which is consistent with the literature review guidelines [27].

## 3. Confidence in Vaccines’ Safety and Efficacy

It is expected that the fear of disease or its complications or knowing someone who died of SARS-CoV-2 will positively impact the vaccination decision and act as a trigger toward safety-related actions. Moreover, official publications of the European Medicines Agency [28] and the Ministries of Health within the countries included in our study [29] are reassuring regarding the local or general adverse events and safety of COVID-19 vaccines.

Unfortunately, fear of unknown long-term side effects (Poland—41.1% of the respondents [30]), side effects (Russia—59.8% [31]; Poland—48.4% [30]; Czech Republic [32]), and anaphylaxis or other serious allergic reaction (Poland—33.2% [30]) due to insufficient and inappropriate scientific studies (Poland—[33]) and concerns about the effectiveness or safety (Russia—61.5% [31]; Romania—40% [34,35]; Czech Republic [32]) of the COVID-19 vaccine [19] threatened the “safety” level from Maslow’s pyramid [36,37,38,39,40].

As far as the confidence in COVID-19 vaccines is concerned, the studies published so far in Eastern European countries reflect (1) the preference for certain vaccine brands, (2) the amplitude of distrust in the clinical trials supporting COVID-19 vaccines, (3) whether with the time and experience people’s attitude changed after vaccine initiation, and (4) the extent to which spirituality, religion, conspiracy beliefs, misinformation, and social relations influence the attitudes toward vaccination.

### 3.1. Vaccine Brand Preferences

Messenger ribonucleic acid (mRNA) preparations have the best reputation. Pfizer BioNTech was indicated in Poland as a first choice by 48% of the respondents, while 9.6% opted for Moderna. Johnson & Johnson was chosen by 6.1% of the respondents, and AstraZeneca would be an option for only 0.6% of the surveyed persons. The type of formulation did not matter for 35.6% of the respondents [33].

In another Polish study, among three COVID-19 vaccines available on the market in Europe at the time of the study, both the Pfizer BioNTech and Moderna vaccines gained a high level of trust in the surveyed group, while the trust in the AstraZeneca vaccine was significantly lower [30].

In Bosnia and Herzegovina, willingness to be vaccinated was reported by 66.4% of subjects, from whom 58.5% preferred Pfizer-BioNTech, 18.8% Sputnik V, 8.7% Sinovac, 7.6% AstraZeneca-Oxford and 6.4% Moderna as an option for vaccination [41].

Despite clearly marked preferences published so far, further studies are needed to include other European reports, explore variables contributing to the acceptance or refusal of each type of vaccine, and assess the level of public understanding of the various vaccines. This understanding would aid policymakers in developing appropriate educational materials to boost confidence in various vaccine platforms. Acknowledging individual preferences for vaccine choice and providing reliable and scientific resources on the various vaccines may tackle vaccine hesitancy and increase vaccine uptake.

### 3.2. Confidence in Trials Evaluating COVID-19 Vaccines and in Resulting Clinical Evidence

The short-term results of clinical trials of different COVID-19 vaccines used in Eastern Europe, excepting Russia, demonstrated their high efficacy against symptomatic SARS-CoV-2 infection, later confirmed by real-world observations. However, in a study conducted in Romania, the majority of those who refused vaccination (63%) thought the COVID-19 vaccine was far too new and that more research was needed to validate it [42].

As opposed to the rest of Eastern Europe, Russia has used a variety of vaccines, but ones that were not backed up by proper evidence on phase III clinical studies. In February 2021, only the phase III efficacy of the Sputnik V vaccine was reported (91.6 percent) [31].

At the same time, studies, including randomized clinical trials, demonstrate that additional doses increase individuals’ benefits in terms of immune response and associated protection [43,44]. In Poland, a study aimed to assess the attitudes of adults who were fully vaccinated toward a potential booster COVID-19 vaccine dose [43]. Overall, 4.3% of the surveyed participants were unsure about receiving the potential booster dose of the COVID-19 vaccine. Two of the most fundamental reasons cited against it were the opinion that a booster dose is unnecessary (39.5%) and concerns about safety (22.4%) [43], despite reassuring published studies data.

### 3.3. Confidence Dynamics in Time: Comparisons before and after Vaccine Initiation or Social Popularization

A comparison of responses collected before and after the start of the immunization campaign in Poland revealed a small increase in the willingness to be vaccinated against COVID-19 (50.7% vs. 51.9%), while the percentage of people who are afraid of the complications after the vaccination has not decreased significantly (51.8% vs. 47.5%). The concern related to the ineffectiveness of vaccination has significantly increased (21.2% vs. 27.6%) [33]. In another Polish study, the number of respondents who expressed concern about the potential adverse events of the COVID-19 vaccination decreased significantly between January and March 2021 (from 76.7% to 66.2%; *p* < 0.01) [19]. According to a study of Facebook comments in Poland, the percentage of positive comments regarding COVID-19 vaccination has increased from 7% to 22% after the first episode of immunization [45].

Similarly, in Russia the acceptance rate increased with verified safety and effectiveness (from 41.7% to 63.2%) [31].

Interesting results were acquired in an experiment in which participants were given positive, compelling messages regarding COVID-19 vaccines before being asked about their vaccination attitudes. Nearly 45% of the responders were unwilling to be vaccinated, and none of the popular, persuasive messages effectively reduced this percentage of hesitancy [46].

It is important to note that despite the passage of time and the experience gained from the various vaccination campaigns conducted so far, and regardless of slight differences in attitude at different moments in time, it seems that the effectiveness of the current information system in transmitting knowledge about vaccination’s safety and efficacy is limited, indicating the need for further improvements.

### 3.4. Associations with Spirituality and Religion

Religiosity and spirituality have been explored to date as influencing general vaccination attitudes.

In a study from the Czech Republic, spiritual respondents were more likely (a 37% increase in the odds) to refuse vaccination [47]. Spiritual but non-religiously affiliated respondents were about 4.43 times more likely to refuse the vaccination than nonspiritual nonreligious respondents. The results indicated that religious affiliation was not associated with any vaccine attitude [47]. Similarly, Polish respondents not affiliated with religious practices had significantly higher odds of refusing the COVID-19 vaccination than the general population [48]. Conversely, another Polish study reported that religiosity correlated with a decrease in vaccine acceptability [49]. However, the last two studies did not clearly define religiosity and did not differentiate religiosity from spirituality.

The results so far are inconclusive, although they suggest a meaningful association between spirituality, religiosity, and attitudes toward COVID-19 vaccination. More studies are needed to increase the level of evidence and deepen the understanding of the factors that might influence the development of religious conspiracy theories in Eastern Europe. Addressing spiritual issues may decrease vaccine refusal and contribute to the effectiveness of the vaccination process.

### 3.5. Conspiracy Beliefs

During COVID-19 pandemics, different pseudoscientific information regarding vaccines was spread. Unverified content proliferated rapidly through social media [50]. An official report in Montenegro, published with the support of the British Embassy and UNICEF [51] concluded that:

*“3 out of 5 citizens in Montenegro believe that there is a secret group of powerful individuals who control global events and that the coronavirus was produced and spread intentionally as a biological weapon so that the world powers could benefit from it in political or economic terms. Furthermore, over one-half of the surveyed citizens believe that the world’s elites created the coronavirus to make the world economies collapse, from which they would benefit financially, and to decrease the number of people on the planet. On the other hand, almost two-thirds of the surveyed citizens do not believe the claim that coronavirus does not exist but rather that it is a result of a conspiracy by the world’s elite to deprive us of our freedom.”*

Pseudoscientific “news” about the COVID-19 vaccine such as its being potentially harmful, its modifying humans’ DNA, its inability to prevent infection, and a supposed 5G chip insertion are associated with vaccination refusal [50]. In Slovenia, many people claimed that the virus had not yet been isolated [52].

A study conducted in Romania reported a correlation between lower levels of conspiracy beliefs (CBs) and higher levels of vaccination approval. The connection between risk perception and inclination to vaccinate was mediated by CBs to a certain degree (β = 0.03, bootstrapped 95% CI: [0.02, 0.05]). Higher levels of CBs were associated with lower risk perception related to SARS-CoV-2 infection, and in turn, lower risk perception was associated with the lack of willingness to vaccinate. Older participants, in particular, were found to have considerably greater COVID-19 risk perception [53]. Given the strong link between risk perception and age, our findings underline the importance of focusing health and communication programs primarily on younger people.

Belief in COVID-19 conspiracy theories (F [4402] = 35.2, *p* < 0.001) was shown to be the most consistent predictor of health behaviors (adherence to the COVID-19 guidelines, usage of pseudoscientific methods, and intentions to receive the vaccine) in a study of 754 Serbians [50]. A very close link existed between CB and a refusal to get the COVID-19 vaccine. Even when the overall conspiracy theory measure removed the vaccine conspiracy theory item and was based on hypotheses regarding the genesis of the virus and political misuse of the health crisis, which should not necessarily impact vaccination intention, this result persisted [50].

These findings emphasize the importance of regional health policies and government information programs in actively advocating against the rising prevalence of CBs about the pandemic and vaccines. The most important action to reduce the spread of CBs is to combat misinformation.

### 3.6. The Misinformation Phenomenon

According to a survey conducted in Poland, the Internet is the primary source of vaccination knowledge for 78% of respondents, followed by healthcare workers other than medical professionals for 38.7%, medical professionals for 38.3%, television for 26.6%, friends for 19.1%, information leaflets for 18.2%, and other sources for 21.1% [33].

The Internet now has a tremendous influence on societal decisions, with 78% of Internet users making vaccination decisions based on information available there. Even a few minutes of exposure to anti-vaccine information on the Internet can have a detrimental impact on people’s perceptions of vaccination risks [54].

Anti-vaxxers propagate disinformation and misleading assertions through social media. Arguments against COVID-19 vaccinations were examined on Facebook in Poland [45]. Only 15% of the comments were pro-vaccination, while 85% were against. The most common anti-vaccine reasons in the dataset were: lack of confidence in the government, vaccine risks, and lack of faith in the availability of an effective vaccine [45]. The risks of vaccines are made unrealistically threatening when emotive storytelling and fear-related facts pique the audience’s interest. Other allegations contend that vaccinations were created only for the benefit of pharmaceutical companies rather than for the sake of society’s health. This argument is intriguing in light of AstraZeneca’s disclosure that the COVID vaccine would not be profitable.

Anti-vaccine social media is known for presenting unsubstantiated information, and COVID-19 vaccine–related misinformation may reduce the intention to vaccinate, impacting vaccination coverage and rates.

### 3.7. The Influence of the Community in Shaping Personal Opinions

In a Romanian study, the pro-vaccination attitude strongly correlated with the subjects’ perception that their primary group accepts vaccination and even correlates with the general public’s perception of pro-vaccination [55]. Therefore, the vaccination decision appears to be closely linked to the social relations system and the rules of the community in which the subject lives.

Moreover, the influence of the social environment was one of the most critical factors associated with vaccination intention in a study conducted in Slovenia, Poland, and Serbia [56].

Acknowledging the particularities of the social relations system may give excellent direction in conducting communication campaigns to popularize the vaccine.

## 4. Health and Vaccine Literacy

Essential concepts that could help increase vaccine acceptance levels are health and vaccine literacy (VL). Access to, comprehension of, and use of health-related information are all part of health literacy [57], which may be characterized on the basis of one’s personal, cognitive, and social abilities. The idea and definition of health literacy are intertwined with health education, which aims to improve a person’s capacity to comprehend and, when the time comes, make effective use of health information. Health literacy is the foundation of vaccine literacy (VL). To put it another way, it’s a degree of understanding the concept of vaccination and the establishment of a system that would assist the transmission or distribution of messages about the necessity of vaccination, without which a working health system would be impossible [57].

### 4.1. Vaccine Literacy in the General Population

A study that evaluated COVID-19 VL in the Croatian adult population shows an average level of VL (M = 2.37, SD = 0.54) [58]. However, the level of COVID-19 VL significantly increased with the level of education (t = 2.453, *p* = 0.032).

In Bosnia and Herzegovina, a study reported that the vast majority of the subjects were not knowledgeable about the COVID-19 vaccination, with correct answer rates going as low as 3.8% on some questions [41]. Higher knowledge regarding COVID-19 and its vaccination was determined as an independent predictor for vaccinating (OR = 23.09, 95% CI: 11.94–44.68) [41].

There is room for progress in the COVID-19 VL level for the adult population through intensified education. Improving the level of COVID-19 education will have an essential role in determining people to accept the vaccine.

### 4.2. Attitudes toward Vaccination in Health Workers or Students

Several studies assessed the willingness to get a COVID-19 vaccine among health workers or medical students, categories of the population less susceptible to misinformation. It is vital to assess the attitudes of medical personnel toward COVID-19 vaccines as the opinions of medical staff influence the opinion of the general population.

The attitudes of physicians toward vaccination were studied in Romania, Poland, Slovenia, and Slovakia. Two-thirds of the Romanian physicians who responded to a questionnaire (61%) would agree to vaccination, while only 2% would not agree, and 27% of respondents would hesitate to accept a new vaccine [59]. An analysis of doctors’ feelings in Poland indicates that 96% of the respondents agree that taking the vaccine will stop the pandemic, but only 58.4% of the respondents consider the vaccine effective [60].

Most Polish ophthalmology residents (71.4%) answered that they would agree to get vaccinated, 17.5% were undecided, and 11.1% said they would not get vaccinated. A willingness to wait until the effectiveness and long-term adverse effects of the vaccine had been assessed was the most common reason for not vaccinating against COVID-19 (72.2%). Other reasons were the belief that the vaccine was not adequately tested (58.3%), fear of complications (41.7%), and the belief that having already contracted COVID-19 protects one from further infections and that there is no longer a need to be vaccinated [61].

In Slovenia, physicians and medical students have a higher intention to get vaccinated, while nurses and technicians were more hesitant [52]. In Slovakia, physician job type (OR = 1.77; 95% CI: 1.13–2.78) was significantly associated with COVID-19 vaccination acceptance. Non-physician healthcare workers (HCWs) distrusted the efficacy of the vaccines significantly more [62].

Similarly, medical students appear to have high acceptance rates and non-negligible levels of hesitancy. In Romania’s largest university of medicine and pharmacy, 88.5% of the students were pro-vaccination, 7.8% were undecided, and 3.7% were vaccine-resistant [63]. Concern about long-term adverse reactions was present in 11.5% of the respondents and significantly more frequent in the undecided and vaccine-resistant [63]. Among Slovak medical students, 22.4% were concerned about severe side effects from the COVID-19 vaccine, and 38.8% were concerned that the COVID-19 vaccine may not be effective [64].

Among nursing students from the Czech Republic, an alarming 21.4% agreed to accept a COVID-19 vaccine, a lower proportion compared with those from Italy (71.2%), Spain (64.6%), Greece (275, 58.5%), and Cyprus (43.5%). The most important reason for the refusal of a COVID-19 vaccine was doubts about the safety, efficacy, and effectiveness of the vaccine (72.4%) [65]. In Poland, most nursing students (77.2%) were vaccinated against COVID-19 [66]. Every other person in the non-vaccinated group declared his or her intention to get a vaccination. Importantly, easy access to vaccines is the most critical factor impacting Polish nursing students’ positive attitudes toward COVID-19 vaccination [66].

Several studies assessing vaccination attitudes among all HCWs were conducted in Eastern Europe. In a study of the employers of a tertiary care hospital in the Czech Republic [67], physicians represented almost a quarter of vaccinated respondents, which is about 14% more than among the unvaccinated. The share of non-healthcare workers predominated in the group of the unvaccinated. Six COVID-19 hospitals and two major universities in Romania undertook a cross-sectional study of HCW [42]. The vaccination rate was 70.42% among the 1021 eligible respondents. Eighteen percent declined immunizations despite having adequate comprehension and knowledge of transmission and treatment of the SARS-CoV-2 infection. At a rate of 88.46%, medical practitioners were the most vaccinated, followed by midwives (68.29%) and pharmacists (63.51%). Nurses (66.03%), social workers (57.57%), and paramedics (50%) all had lower vaccination rates than did medical students (70.41%) [42].

Results confirm that, in general, physicians and medical students have higher vaccination rates and lower hesitance to get vaccinated than non-physician HCWs and nursing students. However, the findings show that vaccine hesitancy persists in medical and social personnel, even among physicians, and, hence, this may be reflected in the hesitancy of the general population toward vaccination. Urgent communication and educational strategies to increase the rate of positive attitudes toward vaccination among HCWs should be integrated in public health measures.

## 5. Confidence in the Healthcare System, Government, and Public Health Measures

Trust in institutions was one of the most critical attitudes associated with vaccination intention and with advising vaccination in a study conducted in Slovenia, Poland, and Serbia [56]. Results from a study conducted in Slovenia demonstrated the positive association between confidence in official sources (experts, public health institutions) and the intention to get vaccinated [52]. According to a study in Poland, the amount of confidence in physicians and science was linked to similar changes in views regarding vaccination [68].

The healthcare system in Russia, on the other hand, was distrusted by 53% of those polled. There were no reports of factors like “the vaccination was advised by a reputable doctor” influencing their choice [31].

Although the World Health Organization (WHO) is viewed as a more trustworthy source of information than the national health ministry or local health department of most countries, there are a few exceptions: Poland (68% for WHO vs. 56% and 51% for national health ministry and local health department, respectively), Russia (63% vs. 40% and 29%), Ukraine (59% vs. 50% and 35%) [69].

Many anti-vaccine arguments are linked to distrust in the government in Poland, according to another study [45].

The lack of confidence in officials has an equally harmful effect.

There was an eight-fold increase (OR = 8.01; 95% confidence interval: 3.65–17.60) in the likelihood of respondents refusing the COVID-19 vaccine when they said they were likely to vote for one of Poland’s right-wing political parties actively supporting the anti-vaccination campaign [48,70].

Immunization reluctance can only be overcome by building people’s faith in the health care system and in the institutions that administer it [71]. People who have a low opinion of experts are more prone to reject scientific consensus and support conspiracy theories that contradict it.

## 6. Predictors of Willingness/Unwillingness to Vaccinate

The health belief model is one of the most commonly used theories in health and illness behavior studies. It is composed of three primary constructs: the perceived benefits (an individual’s beliefs about vaccination), the perceived barriers (the belief that access to vaccination is restricted based on social, environmental, and economic factors), and cues to action (stimuli that motivate an individual to get vaccinated). This model has been demonstrated as an essential predictor of receiving COVID-19 vaccines in Russia [31]. Moreover, females in Russia predicted a more vital willingness to accept the COVID-19 vaccine than did males [71].

The 5C model of vaccine hesitancy, which evaluates individual determinants such as confidence, complacency, convenience, risk calculation, and collective responsibility, was used in Poland to better understand the multidimensional factors that cause vaccine hesitancy or acceptability [49]. Fear of vaccination side effects, confidence in conspiracy theories, and physical fitness were all factors that predicted vaccine refusal [49]. Another Polish cross-sectional survey reported that the lack of higher education of those living in rural areas was significantly (*p* < 0.001) associated with greater odds of refusing the COVID-19 vaccination [48]. Moreover, Poland’s education and population density were positively related to low vaccine hesitancy, while markers of social exclusion, both external (the employment rate) and psychological, affected it negatively [70]. According to a Polish study, factors that influence whether or not people are willing to get immunized include past compliance with vaccination recommendations (OR = 2.082, 95% CI: 1.453–2.982, *p* < 0.001), fear of contracting COVID-19 (OR = 1.560, 95% CI: 1.429–1.701, *p* < 0.001), fear of passing on the disease to relatives (OR = 1.306, 95% CI: 1.219–1.398, *p* < 0.001), and depression symptoms from the previous week (OR = 1.050, 95% CI: 1.011–1.089, *p* = 0.011). When concerns of adverse effects from the vaccine increase, vaccination preparedness decreases (OR = 0.564, 95% CI: 0.531–0.598, *p* < 0.001) [72]. Moreover, older age (<50 vs. ≥50) was a significant factor for vaccine acceptance in Poland [71].

In a Romanian study, the variables that were significantly associated with reporting COVID-19 vaccine acceptance were: living in an urban area (OR = 1.58, 95% CI: 0.98–2.56), being female (OR = 1.59; 95% CI: 1.03–2.44), and being a medical doctor (OR = 3.40; 95% CI: 1.84–6.26) [42].

Higher probabilities of vaccine acceptance were predicted by the degree of confidence in the pharmaceutical sector and healthcare providers and by the perceived knowledge sufficiency, in a university student population, in the Czech Republic [40].

Interestingly, younger individuals in Poland and Russia were more likely to indicate willingness to accept an employer’s vaccination recommendation [71]. Thus, promoting company-provided vaccination campaigns in these countries could positively impact motivating potentially vaccine-hesitant employees.

Determining the most important predictive variables specific to each country or region paves the way to better, targeted educational campaigns aimed at increasing the rates of COVID-19 acceptance.

## 7. Conclusions

Our review regarding hesitancy toward COVID-19 vaccination and its determinants in Eastern Europe showed that individual perceptions play a significant role in the decision to vaccinate. The exposure to misinformation, amplified by the media, the community, and the health and political system, models these perceptions. In order to improve COVID-19 vaccination reach it is necessary to acknowledge and address concerns at every level. Consolidating vaccine literacy is an important tool for combating misinformation. Governments should work closely with communities in a global effort to develop solutions for improved vaccine uptake.

## Supplementary Materials

The following supporting information can be downloaded at: https://www.mdpi.com/article/10.3390/vaccines10050672/s1, Table S1: The main characteristics of the included studies.
