# Supplementary material for: Determinants of the Hesitancy toward COVID-19 Vaccination in Eastern European Countries and the Relationship with Health and Vaccine Literacy: A Literature Review
Source: Vaccines (Basel). 2022 Apr 23;10(5):672. doi: 10.3390/vaccines10050672 (PMC9146656; doi:10.3390/vaccines10050672)
Supplement: Supplementary file 1 [file vaccines-10-00672-s001.zip › vaccines-1659988-supplementary.pdf]

Table S1. The main characteristics of the included studies.

No	Authors	Period	Country	Population	Sample	Data collection	% people intend to get a vaccine	Findings
**Confidence in vaccines's safety and efficacy**								
1	Raciborski F	January and April 2021	Poland	General population	NA	Cross-sectional surveys	31.3% of individuals declared a lack of willingness to vaccinate against COVID-19 regardless of the study wave	- Statistically significant decrease in the percentage of respondents who declared concerns about the potential side effects of the COVID-19 vaccine (from 76.7% to 66.2%; $p < 0.01$)
2	Rzymski P	February and March 2021	Poland	General population	1020	Cross-sectional online survey	NA	- The general trust in different types of vaccines<br>- The level of acceptance of the COVID-19 vaccines already in use in Poland<br>- Level of fear of vaccination against COVID-19<br>- Main sources of information on COVID-19 vaccination
3	Tran VD	September 26th to November 9th, 2020.	Russia	General population	876	Cross-sectional online survey	41.7%	Predictors of the intent to COVID-19 vaccinate:<br>- Believing that the vaccine reduces the risk of virus infection and relieves the complications of the disease<br>- Being unconcerned about side effects and the effectiveness and safety of the vaccination
4	Štěpánek L	30 April - 30 June 2021	Czech Republic	Individuals (n = 7480) who had received at least one dose of the	3224	Cross-sectional surveys	95%	- To protect family members (76.2%),<br>- To prevent the spread of COVID-19 (72.3%)

				COVID-19 vaccine				- Concerns about SARS-nCOV2 (49.1%)<br>- Exemptions from anti-epidemic measures (36.8%)
**Vaccine brand preferences**								
5	Babicki M	14-27 December 2020/19 March 2021	Poland	General population	1559	Online cross-sectional survey	27.1% get vaccinated 50.7% Dec/51.9% March	- Slight increase in the willingness to get vaccinated against COVID-19 over time<br>- The percentage of people who are afraid of the complications after the vaccination has not decreased significantly<br>- Concern related to the ineffectiveness of vaccination has dramatically increased
6	Šljivo A	NA	Bosnia and Herzegovina	General population	570	Cross-sectional	36 (6.3%) were currently vaccinated against COVID-19	- 264 (46.3%) subjects agreed that COVID-19 vaccination programs will prevail in a battle versus COVID-19<br>- Higher knowledge regarding COVID-19 and its vaccination was determined as an independent predictor for vaccinating itself against COVID-19
**Confidence in trials evaluating COVID-19 vaccines and in resulting clinical evidence**								
7	Manolescu LSC	March until July 2021	Romanian Medical and Social Personnel	Romania	1025	Cross-sectional	70.42% vaccinated	- No associations between willingness to vaccinate and vaccine/virus knowledge
8	Rzymski P	September 2021	Poland	General population	2427	Cross-sectional	71% of participants declared willingness to receive a booster COVID-19 dose	- 22.4% expressed concerns about the safety of booster dose
**Confidence dynamics in time: comparison before and after vaccine initiation or social popularization**								

9	Wawrzuta D	NA	Poland	General population	1000	Facebook comments	Pro-vaccine comments increased from 7% to 22% during the event of the first vaccination	Determinants of negative comments on COVID19 vaccination:<br>- Lack of trust in the government<br>- COVID-19 vaccines are dangerous to health<br>- Restriction of free choice<br>- Developed too quickly and without a proper test<br>- Vaccines’ side effects
**Association with spirituality and religion**								
10	Kosarkova A	April 2021	Czech Republic	General population	459	Cross sectional	10%	- Associations of spirituality and religious fundamentalism with religious conspiracy theories about COVID-19 vaccination
11	Raciborski F	8 and 18 April 2021	Poland	General population	1131	Cross-sectional study	¤ 70%;	The factors associated with a lack of willingness to vaccinate against COVID:<br>- People who declared support for one of the right-wing political parties<br>- Rural areas<br>- Lack of higher education
12	Sowa P	12 and 23 of March	Poland	General population	1000	Cross sectional	vaccine hesitancy - 49.2%	Determinants of vaccine hesitancy:<br>- Fear of vaccines' side effects<br>- Beliefs in conspiracy theories<br>- Physical fitness
**Conspiracy beliefs**								
13.	Teovanovic P	10-22 April 2020	Serbia	General	N = 407	Direct questionnaire	49.1% (n = 200)	Predictors of vaccine hesitancy:<br>- Stronger beliefs in COVID-19<br>- Conspiracy theories<br>- Higher levels of COVID-19 related knowledge overestimation<br>- Higher susceptibility to cognitive biases
14	Petravić L	December 2020 to	Slovenia	General population	12,042	Online survey	59%	Estimate the factors that impact the decision of vaccination:<br>- Relatives hospitalized or died due to the virus

								- Healthcare workers
15	Maftei A	January 2021	Romania	General population	247	Cross sectional	40% expressed total rejection of vaccine	- Conspiracy beliefs mediated the relationship between threat perception and willingness of participants to vaccinate
16	Walkowiak MP	30 June and 31 August 2021.	Poland	General population	NA	Longitudinal	increased vaccination rate from 44.2% by an additional 6.6%	- Negative relationship between vaccination rate and conspiratorial views, religiosity, social deprivation and education
17	Riad A	NA	Czech Republic	Nonmedical university students	1351	Cross-Sectional	73.3%	Predictors of vaccine acceptance:<br>- Trust in the pharmaceutical industry<br>- Trust in healthcare providers,<br>- Perceived knowledge sufficiency Predictors of vaccine hesitancy:<br>- Media and social media,<br>- Personal beliefs,<br>- Immunity misconception,<br>- Previous COVID-19 infection,
**The influence of the community in shaping personal opinions**								
18	Cristea D	April 2021	Romania	General population	1001	Cross sectional telephonic interview	40.1% pro vaccination	- Social dimension influenced the willingness to vaccinate
19	Kregar Velikonja N	12 February - 5 March 2021	Slovenia, Poland, and Serbia	Nursing students	872	Cross-sectional study	35%	Vaccination predictors:<br>- Benefits of the vaccine<br>- Trust in institutions<br>- Perceived effectiveness of the vaccine influence of the social environment
**Health and vaccine literacy**								
**Vaccine literacy in general population**								
20	Gusar I	15 to 31 January 2021	Croatia	General population	1227	Cross-sectional study		Higher levels of vaccine literacy provided:<br>- Affirmative answers to questions about the production of safe and efficient vaccines

								- The possibility of vaccinating the entire population, and the need to vaccinate children
**Attitudes towards vaccination in health workers or students**								
21	Herdea V	13 -27 of March 2020	Romanian physicians	Romania	319	Cross-sectional study	61% would agree to vaccination	Predictor of vaccine acceptance:<br>- High level of fear related to COVID-19 pandemic
22	Zdziarski K	February 2021	Poland	Medical doctors	149	Online survey		- Responsibility for their own health, care for their relatives and patients<br>- Vaccine will contribute to stopping the pandemic
23	Konopińska J	January 2021	Poland	Ophthalmology residents	126	Online survey	71.4%	- Willingness to wait for the effectiveness<br>- Long-term adverse effects of the vaccine to be assessed (72.2%)<br>- Feeling that the vaccine was not sufficiently tested (58.3%),<br>- Fear of complications (41.7%)<br>- The belief that having already contracted COVID-19 protects them from further infections
24	Ulbrichtova R	30 August 2021 - 30 September 2021	Slovakia	Healthcare workers	1277	Cross-sectional	84.3%) were vaccinated	Predictors significantly associated with COVID-19 vaccination acceptance:<br>- History of COVID-19 (OR = 0.37; CI95 0.26-0.37),<br>- Influenza vaccination (OR = 1.97; CI95 1.12-3.46),<br>- Compulsory vaccination for HCWs (OR = 9.15; CI95 2.92-28.62),<br>- Compulsory vaccination for selected groups (OR = 9.71; CI95 2.75-34.31) were the e
25	Balan A	12 January until 3 March 2021	Romania	Medical Students	1581	Cross-sectional surveys	88.5%	- Perceived knowledge on the vaccines' safety, efficacy, and technology correlated with a pro-vaccine attitude

26	Sovicova M	10 March 2021 - 24 March 2021	Slovakia	Medical students	1228	Online questionnaire	71.7%)	Barriers towards vaccination:<br>- Side effects from the COVID-19 vaccine<br>- Belief that COVID-19 vaccine may not be effective<br>- Needing more information about the COVID-19 vaccine
27	Patelarou E	December 2020-January 2021	Greece, Albania, Cyprus, Spain, Italy, Czech Republic, and Kosovo	Nursing students	2249	Web survey	53.8%	Barriers<br>- no working experience in healthcare facilities during the pandemic ($p = 0.001$),<br>Facilitators<br>- Vaccination for influenza in 2019 and 2020 ($p < 0.001$),<br>- Trust in doctors ($p < 0.001$), governments and experts ($p = 0.012$)<br>- High level of knowledge ($p < 0.001$)<br>- Fear of COVID-19 ($p < 0.001$).
28	Gotlib J	March and April 2021	Poland	Poland Nursing undergraduate students	793	Cross sectional	77.2% were vaccinated against COVID-19	- Universities' role in education about vaccinations and in shaping pro-vaccine attitudes among students
29	Szmyd B	22 and 25 December 2020	Poland	Medical (MS) and nonmedical (NMS)	1971 - 687 (MS) and 1284 (NMS)	Online questionnaire	91.99% MS<br>59.42% NMS	- Medical studies form pro-vaccination behaviors
**Confidence in the healthcare system, government, and public health measures**								
30	Štěpánek L	February-May 2021	employees of a tertiary care hospital	Czech Republic	4553	Cross-sectional surveys	69.8%	The motive for acceptance<br>- The effort to protect family members (84%)<br>Reasons for hesitancy<br>- Concerns about vaccine safety and side effects (49.4%)<br>- Distrust in the vaccine's efficacy (41.1%)
31	Stasiuk K	December 2020	Poland	General population	400	Longitudinal		- A positive belief in the data published on the Internet by unprofessional sources predicted

								a negative attitude towards vaccination
32	Rozek LS	NA	seventeen countries/territories across five world regions	General population	NA	Cross-sectional	Ukraine, 64% vaccine hesitancy	- Confidence in the national health ministry and the local health department is lower than it is in the WHO in Poland (68% vs. 56% and 51%); Russia (63% vs. 40% and 29%); and Ukraine (59% vs. 50% and 35%)<br>- Ukraine is the only country in which the proportion of respondents that expressed trust in medical professionals fell below 80%
33	Goodwin R	January 2021 to April 2021	Hungary, Israel, Japan	General population	3138	Ecological-systems approach	Hungary, 31%	- In Hungary, vaccine willingness was greatest amongst respondents who trusted their government ($\beta = 0.37$, $p < 0.001$)<br>- Multi-group latent class analysis identified allergies in Hungary as a COVID vaccination myth
**Predictors of willingness/unwillingness to vaccinate**								
34	Lazarus JV	June 2020	13,426 people in 19 countries	Poland	666	Online questionnaire	56.3%	- Identification of potential pockets of heightened distrust
				Russia	680		54.9%	
35	Szmyd B	22 December 2020 to 8 January 2021	Poland	Healthcare workers (HCW)	2300	Google Forms survey	NA	- Willingness to get vaccinated related to Depression, Anxiety and Stress Scale-21 (OR = 1.050, 95% CI: 1.011–1.089, p = 0.011).
36	Riad A	21 April 2021 to 15 June 2021	Czech Republic	University students	1351	Self-administered questionnaire	73.3%	- Higher probabilities of vaccine uptake were predicted by the trust in the pharmaceutical sector and healthcare providers, and by the perceived knowledge sufficiency.

37	Lindholt MF	13 September 2020 to 16 February 2021	Hungary, Denmark and France	General population	7500	Self-administered questionnaire	Hungary, 47%	- Lack of vaccine acceptability is linked to mistrust in authorities and scientists, to conspiracy theories, and to a lack of concern about COVID-19.
**Studies on distinct population**								
38	Gheorghe AS	April-May 2020	Romania	Oncologic patients	1585	Direct questionnaire	72.18%	- Describe the knowledge, attitude and practices (KAP) related to COVID-19 among Romanian oncological patients
39	Matovina Brko G	July and August 2021	Serbia	Oncologic patients	767	Cross sectional	41%	- Reluctant of receiving vaccine due to fear of side due to side effects
40	Marijanović I	February 2021	Bosnia and Herzegovina	Oncologic patients	364	Cross sectional	41.8% answered "Yes" when asked if they would take the vaccine once it becomes available	- In favor of vaccination, the main reasons reported were fear of getting sick (77.6%), the desire to contribute to herd immunity (57.8%), and trusting the recommendations of health professionals (57.2%).<br>- Main reasons for the patients' vaccination -refusal/indecision were doubts about the results from clinical trials of COVID-19 vaccines (49.1%), concerns about adverse effects (24.5%), and confusion about the various vaccine options (19.8%)
41	Kufel-Grabowska J	November 2020 to February 2021	Poland	Polish patients suffering from various chronic diseases, mainly cancer	836	Cross-sectional study	60.5% intended to vaccinate	- Fear of vaccine complications and lack of belief in vaccine effectiveness
42	Puteikis K	December 2020	Lithuania	Pateints with epilepsy and their caregivers	111	Online cross-sectional survey	46.6% PWE 34.0% caregivers	- Thought it could cause the infection (OR = 0.14, 95% CI = 0.04-0.49)

43	Skjefte M	October 28 and November 18, 2020.	16 countries Russia	Non-pregnant mothers	537	383	Online questionnaire	48% likely to accept COVID vaccine	- Confident in the safety of COVID-19 vaccine post-approval by country's health agencies (OR=15.3, 95%CI 5·96-39·3)
				Pregnant women		154			- Confident in COVID-19 vaccine efficacy post-approval by country's health Agencies (OR=15.2,95% CI34- 31·6)
44	Oroszi B	25 January 2021 to 4 July 2021	Hungary	Cases with confirmed SARS-CoV-2	447966		Calculation of indirectly standardized ratios, classified into deprivation quintiles	NA	- Compared to the national average, people living in the most deprived municipalities had a 38% lower vaccination coverage.
45	Sándor J	Vaccinations until 30 June 2021	Hungary	General population	NA		National Health Insurance Fund database of vaccinations.	NA	- Crude vaccination coverage was significantly lower in deprived populations living in segregated colonies (40.05%, 95% CI 39.87%-40.23%) than in complementary areas (65.42%, 95% CI 65.37%-65.46%)
